# Spontaneous Retroperitoneal Bleeding in a Patient with Systemic Lupus Erythematosus

**DOI:** 10.3390/medicina60010078

**Published:** 2023-12-30

**Authors:** Wei-Hung Chen, Deng-Ho Yang

**Affiliations:** 1 Division of Rheumatology/Immunology/Allergy, Department of Internal Medicine, Taichung Armed-Forces General Hospital, Taichung 411, Taiwan; e60416@gmail.com; 2 Division of Rheumatology/Immunology/Allergy, Department of Internal Medicine, Tri-Service General Hospital, National Defense Medical Center, Taipei 114, Taiwan; 3 Department of Medical Laboratory Science and Biotechnology, Central Taiwan University of Science and Technology, Taichung 406, Taiwan; 4 Institute of Biomedical Science, National Chung-Hsing University, Taichung 402, Taiwan

**Keywords:** systemic lupus erythematosus, bleeding, abdominal pain

## Abstract

*Background and Objectives*: Systemic lupus erythematosus (SLE) is a disease with multiple organ involvement, and spontaneous hemorrhage, especially perirenal hemorrhage, is rare. *Case Presentation*: We report the case of a 19-year-old teenager with SLE who experienced left flank pain and hypovolemic shock. Abdominal computed tomography revealed a large left retroperitoneal hematoma. Recurrent hypovolemic shock occurred despite the transcatheter arterial embolization of the left renal artery. Repetitive abdominal computed tomography results showed active hemorrhage. *Result*: An exploratory laparotomy was used to confirm descending colonic mesenteric artery bleeding, which was resolved. The patient needed temporary regular kidney replacement therapy for active lupus nephritis, which terminated one month after discharge. *Conclusions*: When patients with SLE experience acute abdominal pain, flank pain, or back pain combined with hypovolemia, there is a higher risk of bleeding due to spontaneous hemorrhage, which should be included in the differential diagnosis. Therefore, early diagnosis and adequate emergency intervention are necessary.

## 1. Introduction

Systemic lupus erythematosus (SLE) is a systemic and female-predominant autoimmune disease involving multiple organs such as the kidneys, lungs, heart, skin, brain, and musculoskeletal system. The pathogenesis of SLE involves an abnormal immune response, forming immune complexes and autoantibodies [1,2]. Thrombotic events, such as acute ischemic stroke, transient ischemic attack, and myocardial infarction, are common in patients with SLE [3]. However, bleeding complications are rare. Notably, most complications are secondary to pre-existing tumors [4,5], anatomy or structural vessel abnormalities [6,7,8,9,10,11,12], previous hemodialysis [9,10,12,13,14], and thrombocytopenia or coagulation disorders [13,15,16,17,18,19,20]. In addition, kidney invasion accounts for a large proportion of juvenile SLE, and nearly 30% have nephritis [21]. Therefore, we report an unusual case of a boy with spontaneous abdominal bleeding who initially presented with right-sided flank pain, anemia, and hypovolemia, and was later diagnosed with massive retroperitoneal bleeding using abdominal computed tomography.

## 2. Case Presentation

**Figure 1:**
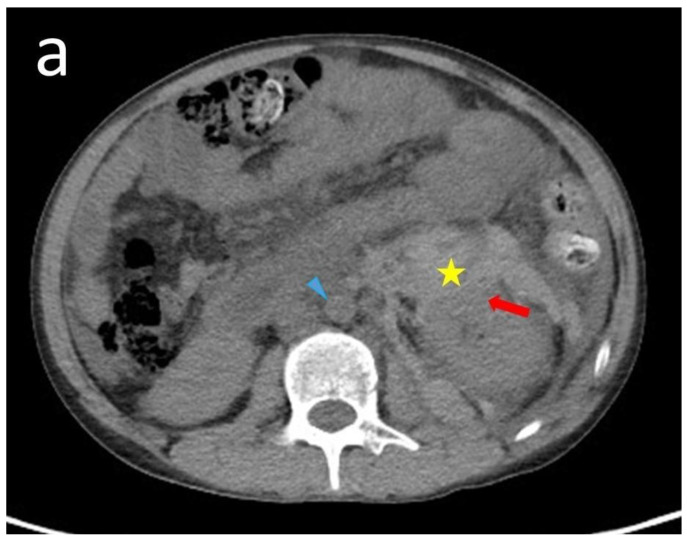

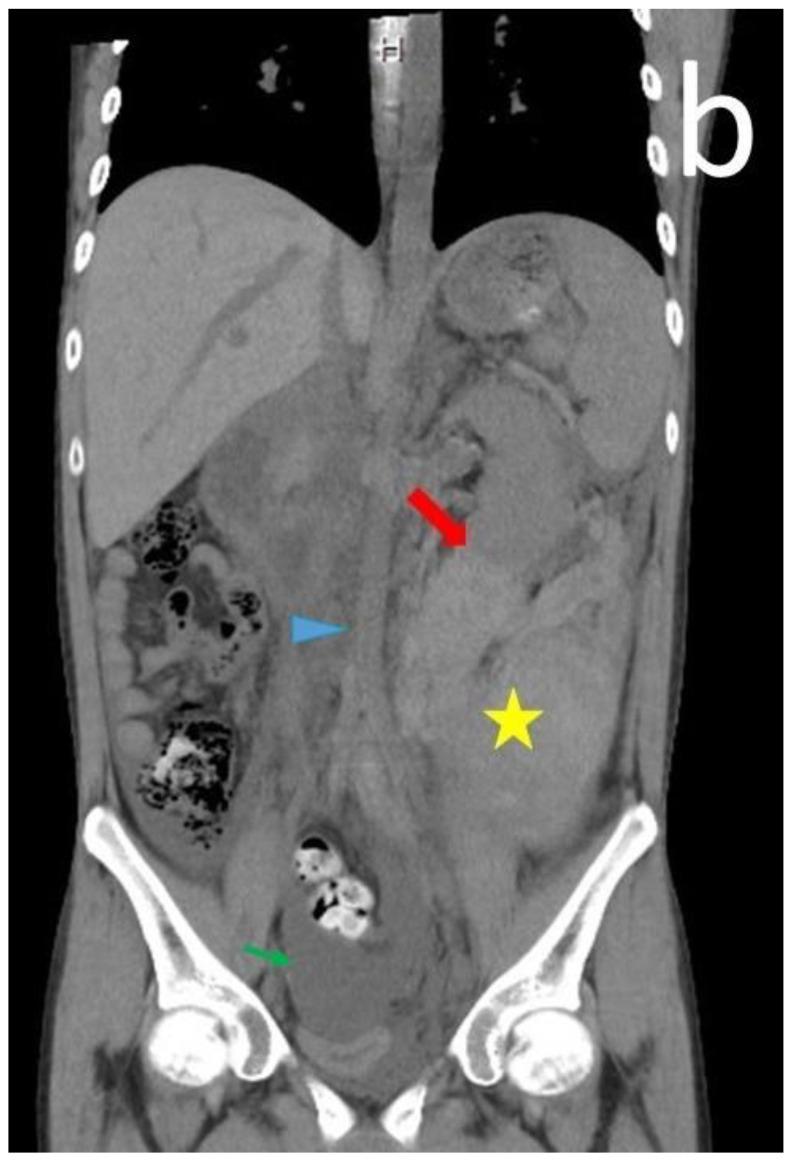
This is a figure showing the CT of the abdomen at the emergency department. Axial view (**a**) and coronal view (**b**). There was a large retroperitoneal hematoma in the left retroperitoneal space (yellow asterisk) and suspected active bleeding in the left kidney lower portion (red thick arrow). Mild ascites (green thin arrow) and decreased volume with a small caliber of the aorta and inferior vena cava (blue arrowhead) were noted.

**Figure 2:**
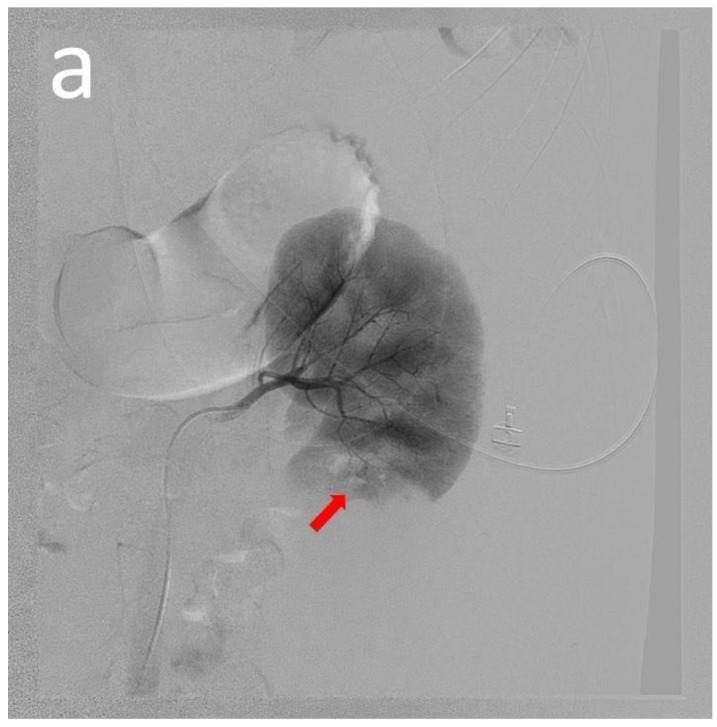

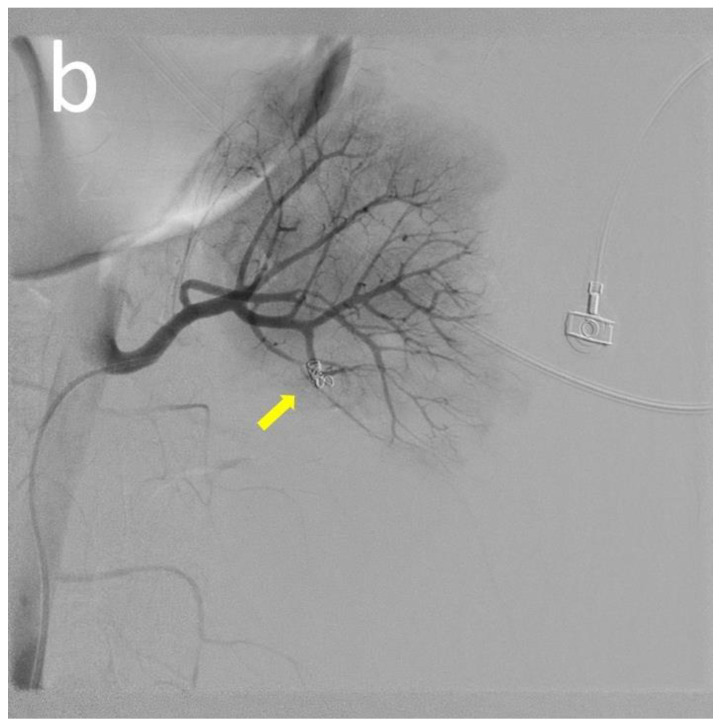
This is a figure showing the transcatheter arterial embolization: The left renal angiography showed minimal contrast extravasation in the left kidney lower portion (red arrow). Angiodysplasia of the left renal artery arteries was found (**a**). Superselective canulation of the tumor-feeding artery arising from the inferior lobar artery of the left renal artery with Terumo microcatheter (Progreat, 2.7 Fr). Two metallic coils (VoetX 3 mm × 3.3 mm, 4 mm × 4 mm) were placed until the sluggish flow of the left renal artery (yellow arrow) (**b**).

A 19-year-old male presented with a 12-year history of SLE and took prednisolone and hydroxychloroquine for long-term control. His mother had SLE and died from it. He presented to our hospital with a 1-day intermittent left flank pain. On visiting our emergency department, his vital signs were a body temperature of 35.0 °C, a pulse of 114 beats/min, a respiratory rate of 18 times/min, a blood pressure of 115/71 mmHg, and a saturation in room air of 100%. He denied having other symptoms, such as fever, chills, dysuria, frequency, urgency, or abdominal pain. Physical examination revealed a pale conjunctiva and left flank knocking tenderness. Laboratory data was as follows: hemoglobin: 6.7 g/dL (normal range: 13–18 g/dL); hematocrit: 19.9% (normal range: 40–52%); blood urea nitrogen: 147.8 mg/dL (normal range: 5–25 mg/dL); creatinine: 1.9 mg/dL (normal range: 0.7–1.3 mg/dL); GOT: 8 U/L (normal range: 0–40 U/L); WBC: 26,600/μuL (normal range: 4000–10,000/μL); platelet: 214,000/μL (normal range: 150,000–450,000/μL); PT: 10.7 s (normal range: 8–12 s); aPTT: 39.7 s (normal range: 23.9–35.5 s); and INR: 1.07 (normal range: 0.85–1.15). Abdominal computed tomography (CT) without contrast (Figure 1a,b) revealed a large hematoma in the left retroperitoneal space, suggesting active bleeding in the lower portion of the left kidney. Transcatheter arterial embolization (Figure 2a,b) showed minimal contrast extravasation in the lower portion of the left kidney and angiodysplasia in the left renal artery. Two metallic coils were placed to ensure a sluggish flow in the left renal artery. During the first few days of hospitalization in the intensive care unit, we checked for autoimmune antibodies, and we recorded positive values for antinuclear antibody (1:160, homogenous pattern, normal range: <1:40), Anti-ds DNA: 78 IU/mL (normal range: <10.0 IU/mL), and weakly positive anti-cardiolipin IgM: 20.5 U/mL (normal range: <10 U/mL). Other autoimmune antibodies of anti-cardiolipin IgG, β2-glycoprotein IgM, β2-glycoprotein IgG, anti-smith, and anti-ribonucleoprotein were negative. Laboratory data showed decreased C3 (64.4 mg/dL, normal range: 87–200 mg/dL) and C4 (10.2 mg/dL, normal range: 19–52 mg/dL) complement levels, increased lupus anti-coagulant levels of 1.23 (normal range: ≤1.2), and increased IgM (>500 mg/dL, normal range: 45–281 mg/dL) levels.

**Figure 3:**
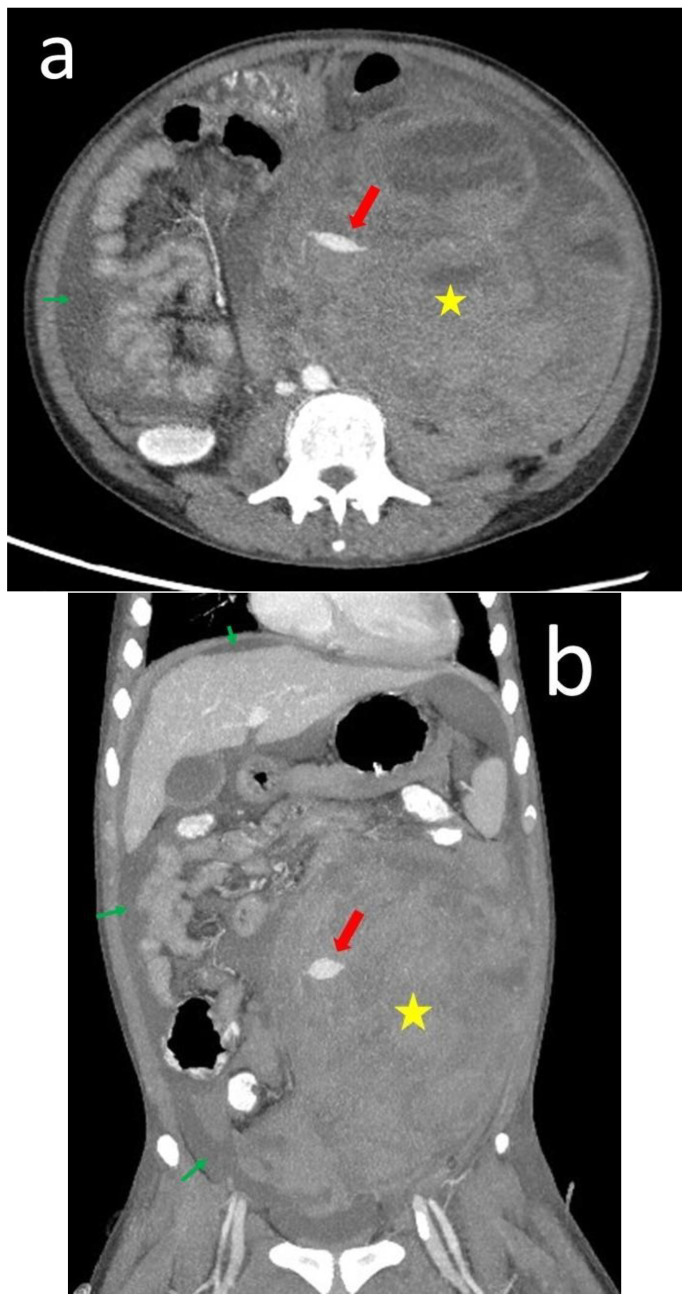
This is a figure showing the CT of the abdomen on day 5 of admission. Axial view (**a**) and coronal view (**b**). There was an active hemorrhage in the left retroperitoneal space hematoma (yellow asterisk) with high-density contrast retention (red arrow). The ascites increased compared with the day of admission (green thin arrow).

Due to the persistently unstable hemodynamic status, we performed abdominal computed tomography angiography and still observed an active hemorrhage in the hematoma with high-density contrast retention (Figure 3a,b). Therefore, the patient underwent an exploratory laparotomy with retroperitoneal exploration. Consequently, a marked ileus, intraperitoneal adhesion, and active oozing of the descending colon mesentery artery were observed, suggesting an inferior mesenteric artery origin. Enterolysis and hematoma evacuation were at approximately 3.9 L. Blood and clot tests were performed. The pathological report showed mixed acute and chronic inflammation with fibrosis without morphological evidence of malignancy.

After surgical intervention, the patient’s renal function improved; however, he developed oliguria and shortness of breath 2 weeks later. A 3-day course of low-dose pulse steroid therapy with 160 mg of intravenous methylprednisolone daily was administered, and the patient subsequently received regular hemodialysis because of no recovery of renal function. Consequently, the patient received an indwelling Hickman catheter for long-term renal replacement therapy and was discharged in stable condition. During the outpatient department follow-up, his blood urea, nitrogen, and creatinine levels improved with appropriate urine output one month later. The Hickman catheter was removed, and long-term hemodialysis was no longer required. Unfortunately, this patient had another episode of acute kidney injury four months after discharge. At that time, cardiac arrest combined with respiratory failure occurred. Consequently, he received a tracheotomy because he needed to be trained to wean off the ventilator, and he was transferred to the respiratory care center of another hospital.

## 3. Discussion

Anemia is observed in almost all patients with bleeding. Nonspecific signs, such as fatigue, dizziness, or tachycardia, may be present, and severe cases may present as hypovolemic shock. Notably, other symptoms are diverse and strongly associated with the bleeding location. Previous case reports have reported several spontaneous bleeding sites, including the gastrointestinal tract, muscle, corpus luteum, brain, spleen, soft tissue, and adrenal gland in patients with SLE [10,11,16,17,19,22,23,24]. One cohort study demonstrated an increased risk of subarachnoid hemorrhage in patients with SLE [25].

Patients with abdominal pain or fullness may have bleeding sites in the abdominal cavity. However, palpable masses or ecchymoses may indicate the corresponding bleeding region. Patients with spontaneous non-traumatic renal hemorrhage (so-called Wünderlich syndrome) may present with the classic “Lenk’s triad” [15], including acute flank pain, flank mass, and hypovolemia. Regarding imaging studies, sonography and computed tomography are indispensable and can accurately detect bleeding events. Consequently, angiography can further determine the bleeding location.

A case report has summarized the possible causes of Wünderlich syndrome [12], including malignancies or neoplasms, cystic renal diseases, vascular events (vasculitis and aneurysm), infectious processes, and bleeding diathesis (systemic anticoagulation and end-stage renal disease). At least one symptom of Lenk’s triad could be found in the case reports we reviewed. Most cases have explanations that correspond to the cause of bleeding: cystic renal diseases [8,9], vascular events [6,7,12,15,26], thrombocytopenia or coagulation disorders [13,15,20], and end-stage renal disease under hemodialysis [9,12,13,14].

The laboratory data and patterns showed a strong relationship between spontaneous renal bleeding and SLE disease activity. Vasculitis-like manifestations are also associated with renal artery hemorrhage. These features include decreased complement level [6,12,15,24,26], positive ANA and/or Anti-ds DNA [6,7,12,14,15,24], antiphospholipid antibodies (anti-Cardiolipin IgG and IgM, and anti-β2-Glycoprotein IgG and IgM), and increased lupus anti-coagulant [24].

The treatment for bleeding varies from conservative blood transfusions, and medication, to vascular embolization and aggressive organ resection. In the cases we reviewed, only three patients underwent organ resection with pathological reports, resulting in hemorrhagic renal cysts, membranoproliferative glomerulonephritis, acute and chronic pyelonephritis, lupus nephritis, necrotic Wilms tumor, and renal cell carcinoma [4,8,26]. In other case reports [6,7,9,12], structural abnormalities were observed on imaging tests; however, all microscopic changes remained unknown because no further pathological specimens were obtained. A summary of previously published cases of spontaneous perinephric hemorrhage associated with SLE is shown in Table 1 [6,7,8,9,12,13,14,15,20,24,26,27].

In this present case, the patient presented with symptoms of Lenk’s triad, except for a palpable flank mass. He was diagnosed with retroperitoneal bleeding based on abdominal computed tomography results. From the transcatheter arterial embolization examination, the initial bleeding site was considered to be angiodysplasia of the left renal artery. Unfortunately, the active retroperitoneal bleeding was not controlled, and another bleeding site in the branch of the inferior mesenteric artery was confirmed using exploratory laparotomy. We obtained no specimens from the kidney or descending colon mesentery artery during the entire procedure, and no further histological changes were observed based on laboratory outcomes. Furthermore, because of no remarkable prolonged aPTT, we unfavored a coagulopathy disorder to be the cause of bleeding. Thus, we did not have relevant tests such as Von Willebrand factor deficiency or other coagulation disorders due to a lack of coagulation factors (factor XIII, factor IX, Coagulation factor inhibitors, factor eight inhibitor bypass activity, and recombinant activated factor VII). This is the limitation of our case.

We speculate that the patient had active lupus nephritis due to the poorly controlled nephrotic syndrome. SLE-related vasculitis results in subsequent spontaneous renal artery bleeding and favored inferior mesenteric artery bleeding.

Based on a previous analysis of the risk factors for Wünderlich syndrome, the only risk factor in our patient was angiodysplasia of the left renal artery, which was not recovered in the past. Unfortunately, hemorrhage occurred, and the location of bleeding was unusual compared with that described in previous literature reviews.

## 4. Conclusions

Patients with SLE and active nephrotic syndrome, bleeding tendency, anatomy, or structural vessel abnormalities may have a higher incidence of spontaneous hemorrhage. If patients present with Lenk’s triad, spontaneous renal bleeding should be included in the diagnosis and can be diagnosed using imaging examinations, such as abdominal sonography and computed tomography. Therefore, early diagnosis and adequate emergency treatment are necessary for these patients.

## Figures and Tables

**Table 1:** Summary of previously published cases of SLE-associated spontaneous perirenal hemorrhage.

No	Study	Age	Gender	History of SLE (Years)	Symptoms	Renal Function	Coagulopathy	Tools for Diagnosis	Bleeding Site	Anatomical Anomalies	Autoantibody Test	Treatment	Pathological Result	Outcome
1	Tsai et al. [6]	21	Male	Recent	Abdominal pain Anemia (Hb: 8.1 g/dL)	Normal	Normal	CT	Multiple small aneurysms	Multiple small aneurysms in the bilateral renal arteries	Anti-dsDNA antibody	Steroid AZA HcQ	N/A	Alive
2	Melamed et al. [7]	36	Male	10	Flank pain Anemia (Hb: 7.4 g/dL)	N/A	Normal	CT	Multiple microaneurysms	Bilateral multiple renal artery microaneurysms	Anti-dsDNA antibody	Steroid CTX	N/A	Expired
3	Lin et al. [8]	46	Female	N/A	Anemia (Hct: 27.5%)	N/A	N/A	CT	Cystic lesion	Cystic lesion at upper pole of right kidney	N/A	Nephron sparing enucleation	Hemorrhagic cyst	Alive
4	Ku et al. [9]	46	Male	N/A	Flank pain with tender palpable mass Anemia	HD (8 yeas)	N/A	CT	Multiple cysts	Multiple cysts	N/A	Nephrectomy	Multiple cysts	Alive
5	Chao et al. [12]	39	Female	20	Abdominal pain Anemia (Hct: 16%)	HD (10 years)	Normal	CT	Small aneurysmal sacs	Small aneurysmal sacs in the right kidney	Anti-dsDNA antibody	Embolization Steroid	N/A	Alive
6	Chang et al. [13]	30	Male	6	Flank pain Anemia (Hb: 7.0 g/dL)	HD (6 years)	Thrombocytopenia	CT	N/A	N/A	N/A	Conservative	N/A	Alive
7	Furkan et al. [14]	30	Female	N/A	Fank pain Anemia (Hb: 7.8 g/dL)	HD (7 years)	Normal	CT	Rupture of the right kidney	no	Anti-dsDNA antibody	Conservative	N/A	Alive
8	Zhao et al. [15]	33	Female	Recent	Abdominal guarding and rigidity Flank tenderness Anemia (Hb: 4.2 g/dL)	Normal	Thrombocytopenia	CT	Distant branch of the left renal artery	No	Anti-dsDNA antibody, Anti-smith antibody	Embolization Steroid HcQ CsA CTX	N/A	Alive
9	Sahin et al. [20]	32	Male	N/A	Fank and abdominal tenderness	N/A	Coagulopathy	CT	Left ureteropelvic junction	N/A	N/A	FEIBA	N/A	Alive
10	Wang et al. [24]	58	Female	1.5	Anemia (Hb: 5.3 g/dL)	N/A	N/A	CT	Adrenal gland	N/A	Anti-dsDNA antibody, Anti-U1-snRNP/Sm Anticardiolipin antibody Anti-β2-glycoprotein Lupus anticoagulant	Conservative NOAC	N/A	Alive
11	Dux et al. [26]	50	Female	N/A	Palpated hypochondrium mass Loin pain Anemia (Hb: 8.1 g/dL)	ClCr: 2 mL/min no HD	N/A	IV pyelography	Lower pole of the kidney	N/A	N/A	Nephrectomy	Tear of 16 mm in the lower pole of kidney, no connection between the site of biopsy and rupture	Alive
12	MISHRIKI et al. [27]	36	Female	12	Left upper abdominal pain	Normal	N/A	CT	Presumed junction between the renal pelvis and kidney anteriorly	N/A	N/A	Nephrectomy Steroid AZA	Postmortem renal biopsy: within normal limits	Expired
13	Our case	18	Male	12	Flank pain Anemia(Hb: 6.7 g/dL)	Normal	Normal	CT	Angiodysplasia of the left renal artery & branch of IMA	N/A	Anti-dsDNA antibody Lupus anti-coagulant Anti-cardiolipin IgM	Embolization Steroid	N/A	Alive

N/A: Not available, Hb: Hemoglobin, Hct: Hematocrit, ClCr: Creatinine clearance rate, HD: Hemodialysis, CT: Computed tomography, IV: Intravenous, C3: Complement 3, C4: Complement 4, ANA: Antinuclear antibody, AZA: Azathioprine, CTX: Cyclophosphamide, CsA: Cyclosporin, HCQ: Hydroxychloroquine, NOAC: Novel oral anticoagulants, FEIBA: Factor eight inhibitor bypass activity, Conservative treatment: blood transfusion, absolute bed rest, fluid resuscitation.

## Data Availability

The data that support the findings of this study are available from the corresponding author, D.-H.Y., upon reasonable request.

## References

[1] D’Andrea D.M., Coupaye-Gerard B., Kleyman T.R., Foster M.H., Madaio M.P. (1996). Lupus autoantibodies interact directly with distinct glomerular and vascular cell surface antigens. Kidney Int..

[2] Vlahakos D.V., Foster M.H., Adams S., Katz M., Ucci A.A., Barrett K.J., Datta S.K., Madaio M.P. (1992). Anti-DNA antibodies form immune deposits at distinct glomerular and vascular sites. Kidney Int..

[3] Sarabi Z.S., Chang E., Bobba R., Ibanez D., Gladman D., Urowitz M., Fortin P.R. (2005). Incidence rates of arterial and venous thrombosis after diagnosis of systemic lupus erythematosus. Arthritis Care Res. Off. J. Am. Coll. Rheumatol..

[4] Mcdougal W.S., Kursh E.D., Persky L. (1975). Spontaneous Rupture of the Kidney with Perirenal Hematoma. J. Urol..

[5] Hellström P.A., Mehik A., Talja M.T., Siniluoto T.M., Perälä J.M., Leinonen S.S. (1999). Spontaneous subcapsular or perirenal haemorrhage caused by renal tumours: A urological emergency. Scand. J. Urol. Nephrol..

[6] Tsai Y.-G., Lai J.-H., Kuo S.-Y., Chen H.-C., Wan H.-L., Chang D.-M. (2003). Ruptured renal microaneurysms complicated with a retroperitoneal abscess for a patient with systemic lupus erythematosus. Lupus.

[7] Melamed N., Molad Y. (2006). Spontaneous retroperitoneal bleeding from renal microaneurysms and pancreatic pseudocyst in a patient with systemic lupus erythematosus. Scand. J. Rheumatol..

[8] Lin Y.-Y., Chen J.-D., How C.-K., Yen D.H.-T. (2010). Spontaneous Perinephric Hemorrhage from a Hemorrhagic Renal Cyst. Intern. Med..

[9] Ku J.H., Kim J.-K., Ha S., Lee J.W. (2009). Bilateral spontaneous perirenal haemorrhage in a patient on haemodialysis. Nephrol. Dial. Transplant. Plus.

[10] Libra F., Falsaperla D., Desiderio C.M., Santo Signorelli S., Palmucci S., Basile A. (2020). Spontaneous bleeding in systemic lupus erythe-matosus: Endovascular treatment of two rare cases. Radiol. Case Rep..

[11] Seong S.-S., Joung C.-I. (2010). A case of spontaneous hemoperitoneum presenting as the initial manifestation of systemic lupus erythematosus. Korean J. Intern. Med..

[12] Chao C.-T., Wang W.-J., Ting J.-T. (2013). Wünderlich Syndrome from Lupus-Associated Vasculitis. Am. J. Kidney Dis..

[13] Chang T.-H., Wu W.-J., Hsiao H.-L., Yeh H.-C., Huang C.-H., Lee Y.-C. (2005). Spontaneous Perirenal Hematoma: A Case Report. Kaohsiung J. Med. Sci..

[14] Ufuk F., Herek D. (2016). Life-threatening spontaneous kidney rupture in a rare case with systemic lupus erythematosus: Prompt diagnosis with computed tomography. Hemodial. Int..

[15] Zhao Y., Jia X., Tong X., Niu G., Wang R., Liu L., Zhou F. (2021). Spontaneous perirenal hemorrhage in systemic lupus erythematosus: A rare case report and literature review. BMC Nephrol..

[16] Yacobovich J.R., Uziel Y., Friedman Z., Radnay J., Wolach B. (2001). Diffuse muscular haemorrhage as presenting sign of juvenile systemic lupus erythematosus and lupus anticoagulant hypoprothrombinaemia syndrome. Rheumatology.

[17] Lu C.-C., Chen C.-H., Yeh S.-F., Lai J.-H., Chang D.-M. (2012). A spontaneous intercostal artery hemorrhage in systemic lupus erythematosus. Rheumatol. Int..

[18] Abdulla M. (2016). Spontaneous soft tissue haemorrhage in systemic lupus erythematosus. Reumatismo.

[19] Pérez M.L., Laso R.V., Velasco-Rodríguez D., Martín-Herrero S., Alfonzo I.M., García-Raso A., Llamas-Sillero P. (2023). Lupus anticoagulant-hypoprothrombinemia syndrome: A cerebral bleeding case report as systemic lupus erythematosus debut. Reumatol. Clínica.

[20] Sahin T.K., Aladag E., Setterzade E., Guven G.S., Haznedaroglu I.C., Aksu S. (2020). Spontaneous subepithelial hemorrhage of renal pelvis and ureter (Antopol-Goldman lesion) in hemophilia a patient with inhibitor: Case report and review of the literature. Medicine.

[21] Sahin S., Adrovic A., Barut K., Canpolat N., Ozluk Y., Kilicaslan I., Caliskan S., Sever L., Kasapcopur O. (2018). Juvenile systemic lupus erythematosus in Turkey: Demographic, clinical and laboratory features with disease activity and outcome. Lupus.

[22] Paul R., Bandyopadhyay R., Santra S., Patra T.K. (2010). A Case of Systemic Lupus Erythematosus with Spontaneous Intracranial Epidural Hematoma. J. Med. Cases.

[23] Itagaki M.W., Gregory J.S. (2005). Spontaneous splenic artery hemorrhage with secondary antiphospholipid syndrome in lupus: A case report. Lupus.

[24] Wang Y., Zhang G., Zhang L., Luo J., Gao L. (2019). Adrenal hemorrhage in a patient with systemic lupus erythematosus. J. Peking Univ. Health Sci..

[25] Chang Y.-S., Liu C.-J., Chen W.-S., Lai C.-C., Wang S.-H., Chen T.-J., Tzeng C.-H., Tsai C.-Y., Wang S.-J. (2012). Increased Risk of Subarachnoid Hemorrhage in Patients with Systemic Lupus Erythematosus: A Nationwide Population-Based Study. Arthritis Care Res..

[26] Dux S., Pitlik S., Boner G., Ben-Bassat M., Rosenfeld J.B. (1982). Spontaneous Rupture of the Kidney in a Patient with Acute Autoimmune Disease. Urol. Int..

[27] Mishriki S.F., Hopkinson N., Shepherd D.F., Parham D.M., Rundle J.S. (1999). Bilateral rupture of the renal pelves associated with systemic lupus erythematosus. BJU Int..

